# Chitosan Nanoparticles Attenuate Hydrogen Peroxide-Induced Stress Injury in Mouse Macrophage RAW264.7 Cells

**DOI:** 10.3390/md11103582

**Published:** 2013-09-30

**Authors:** Zheng-Shun Wen, Li-Jia Liu, You-Le Qu, Xiao-Kun OuYang, Li-Ye Yang, Zi-Rong Xu

**Affiliations:** 1 Zhejiang Provincial Key Engineering Technology Research Center of Marine Biomedical Products, Food and Pharmacy College, Zhejiang Ocean University, Zhoushan, Zhejiang 316000, China; E-Mails: liulijiatu_tu@126.com (L.-J.L.); 77280116@qq.com (Y.-L.Q.); xkouyang@163.com (X.-K.O.Y.); liyey@zjou.edu.cn (L.-Y.Y.); 2 Key Laboratory for Molecular Animal Nutrition of Ministry of Education, Feed Science Institute, College of Animal Sciences, Zhejiang University, Zijingang Campus, Hangzhou 310058, China

**Keywords:** chitosan nanoparticles, antioxidant activity, stress injury, RAW264.7 cells

## Abstract

This study was carried out to investigate the protective effects of chitosan nanoparticles (CNP) against hydrogen peroxide (H_2_O_2_)-induced oxidative damage in murine macrophages RAW264.7 cells. After 24 h pre-incubation with CNP (25–200 μg/mL) and chitosan (CS) (50–200 μg/mL, as controls), the viability loss in RAW264.7 cells induced by H_2_O_2_ (500 μM) for 12 h was markedly restored in a concentration-dependent manner as measured by MTT assay (*P* < 0.05) and decreased in cellular LDH release (*P* < 0.05). Moreover, CNP also exerted preventive effects on suppressing the production of lipid peroxidation such as malondialdehyde (MDA) (*P* < 0.05), restoring activities of endogenous antioxidant including superoxide dismutase (SOD), and glutathione peroxidase (GSH-Px) (*P* < 0.05), along with increasing total antioxidant capacity (T-AOC) (*P* < 0.05). In addition, pre-incubation of CNP with RAW264.7 cells for 24 h resulted in the increase of the gene expression level of endogenous antioxidant enzymes, such as MnSOD and GSH-Px (*P* < 0.05). At the same concentration, CNP significantly decreased LDH release and MDA (*P* < 0.05) as well as increased MnSOD, GSH-Px, and T-AOC activities (*P* < 0.05) as compared to CS. Taken together, our findings suggest that CNP can more effectively protect RAW264.7 cells against oxidative stress by H_2_O_2_ as compared to CS, which might be used as a potential natural compound-based antioxidant in the functional food and pharmaceutical industries.

## 1. Introduction

Antioxidants may have a positive effect on human health since they can protect human body against deterioration by reactive oxygen species (ROS), which attack biomolecules such as proteins, lipid, and DNA, and consequently, lead to many health disorders including aging, cancer, diabetes, neurodegenerative, cardiovascular, and inflammation disease. Cells possess cellular defense systems including nonenzymatic and enzymatic substances, such as superoxide dismutase (SOD) and glutathione peroxidase (GSH-Px) to scavenge radicals and protect major cellular biomolecules from oxidative damage to [1,2,3]. When the cellular defense mechanisms are unable to cope with excessive generation of ROS, subsequently oxidative stress will happen. Oxidative stress has been shown to be involved in various pathogenic processes including aging, cancer, wrinkle formation, rheumatoid arthritis, and inflammation. Therefore, antioxidant might theoretically retard spreading of cell damage and improve intracellular antioxidant enzymatic and nonenzymatic systems.

Many studies have been carried out to seek novel antioxidative compounds from marine resources, such as chitin, chitosan, and their derivates. Chitosan, a partially deacetylated polymer of N-acetyl glucosamine, is prepared by alkaline deacetylation of chitin. It exhibits a wide variety of biological activities, including antitumor activities [4], immunostimulating effects [5], cholesterol-lowering effects [6], antimicrobial effects [7], wound-healing effects [8], antifungal activities, and free radical scavenging activities [9]. Xie, Xu, and Liu (2001) reported that the scavenging of hydroxyl radicals by chitosan inhibits the lipid peroxidation of phosphatidylcholine and linoleate liposomes [10]. Santhosh, Sini, Anandan, and Mathew (2006) reported that the administration of chitosan to rats, treated with isoniazid or rifampicin, prevented the oxidation of hepatotoxic lipids [11]. Similarly, chitosan, when injected, inhibited glycerol-induced renal oxidative damage in rats [12]. Owing to its many antioxidant studies *in vitro* and *in vivo*, chitosan has attracted considerable attention from researchers.

In the previous studies, the antioxidant activity of chitosan, and its oligosaccharides, was studied *in vitro* and *in vivo*. The results showed that chitosan could significantly reduce serum free fatty acid (FFA) and MDA concentrations and elevate SOD, CAT, and GSH-Px activities, the latter being the major antioxidant enzyme in the body, indicating that chitosan regulated the antioxidant enzyme activities and reduced lipid peroxidation [13]. The unique character of nanoparticles could make chitosan nanoparticles (CNP) exhibit more superior activities than chitosan, CNP have been reported to have heightened immune-enhancing effect [14], anticancer activity [15], and antimicrobial activity [16], of chitosan (CS). However, other biological activities are unknown, such as the antioxidant activity of CNP, has received less attention. Therefore, we hypothesized that CNP may have a potential to augment the antioxidant activity of chitosan. Herein, we investigated the protective effects of CNP against hydrogen peroxide (H_2_O_2_)-induced oxidative damage in murine macrophages RAW264.7 cells.

## 2. Results

### 2.1. Morphology, Size, and Zeta Potential of CNP

**Figure 1:**
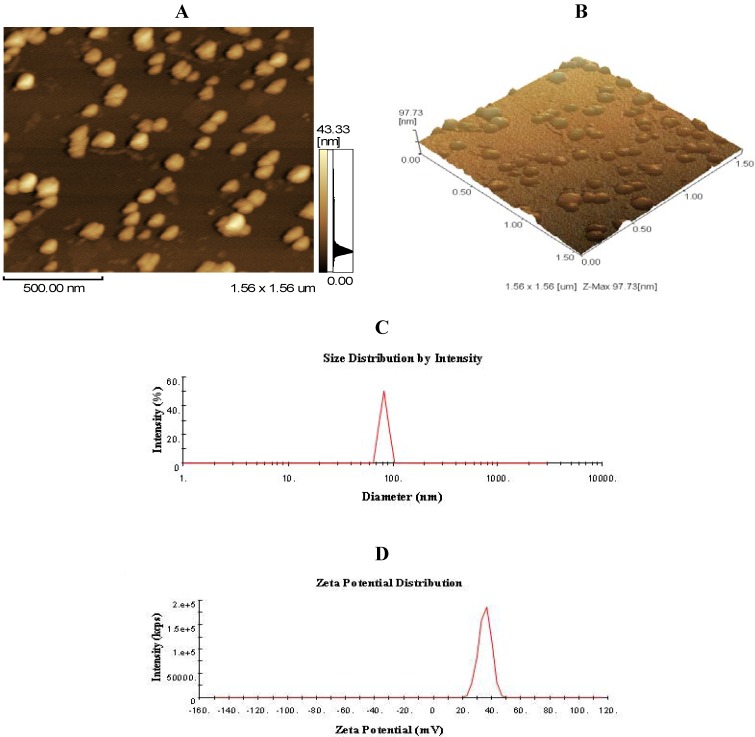
Morphology of chitosan nanoparticles (CNP). (**A**,**B**) atomic force micrographs (AFMs) of CNP; (**C**) the size distribution by intendity of CNP, the size of CNP ranges from 63.16 to 101.70 nm, and the mean of size is about 83.66 nm; (**D**) Zeta potential distribution of CNP, CNP exhibit a zeta potential range from 20.04 to 51.13 mV and have a mean charge with 35.43 mV.

As shown in Figure 1A,B, chitosan nanoparticles regularly formed and well distributed in the acetic acid (HOAc)/sodium tripolyphosphate solution (pH 5.5) used in this study. The mean size and size distribution of each batch of nanoparticle suspension was analyzed using the Zetasizer analysis (Figure 1C,D). The size distribution profile represents a typical batch of nanoparticles with a mean diameter of 83.66 nm and a narrow size distribution ranging from 63.16 to 101.70 nm (polydispersity index < 1), and shows that the surfaces of chitosan nanoparticles have a positive surface charge of about 35.43 mV. These excellent characteristics are of benefit to the stabilization and penetration capability of CNP.

### 2.2. Time-Dependent and Concentration-Dependent Viability Losses in RAW264.7 Cells Induced by H_2_O_2_

**Figure 2:**
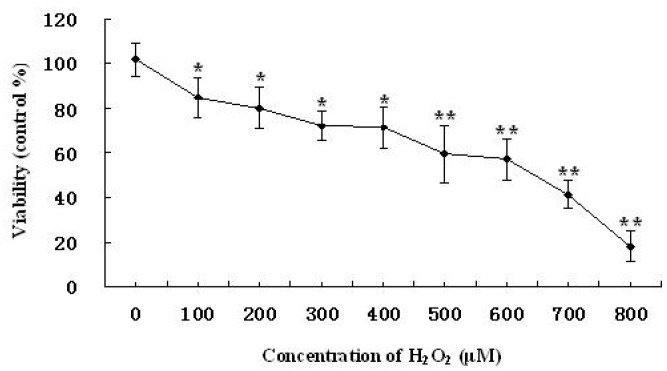
Viability losses in RAW264.7 cells induced by various concentration of H_2_O_2_. Values are presented as means ± SD. Mean with different asterisks are statistically different (*P* < 0.05).

**Figure 3:**
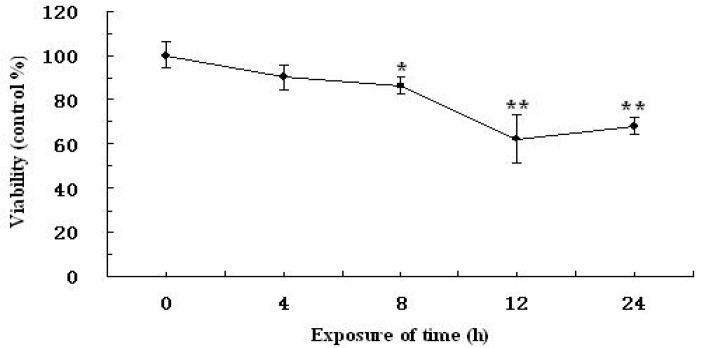
Viability losses in RAW264.7 cells induced by H_2_O_2_ for different time. Values are presented as means ± SD. Mean with different asterisks are statistically different (*P* < 0.05).

The time-dependent and concentration-dependent studies of viability losses were investigated in RAW264.7 cells induced by H_2_O_2_. After treatment with increasing concentrations of H_2_O_2_ for 12 h, cell viability was then examined by MTT method. As shown in Figure 2, gradual losses of cell viability were observed with increasing concentrations of H_2_O_2_. The degree of cell injury was maximum at 800 μM, among the cell viability was close to 18.0% ± 7.0% as compared with the vehicle-treated control group. The results of time-response study in which cells were exposed to 500 μM of H_2_O_2_ up to 24 h (Figure 3), the concentration of H_2_O_2_ started to increase oxidative injury in RAW264.7 cells 4 h after treatment with H_2_O_2._ The magnitude of cell injury peaked at 12 h after H_2_O_2_ exposure, and the cell viability was about 59.1% ± 12.6% (*P* < 0.05). Based on these results, RAW264.7 cells were treated with 500 μM of H_2_O_2_ for 12 h, or vehicle as control, in the extensive studies.

### 2.3. Effect of CNP on the Viability of H_2_O_2_-Induced RAW264.7 Cells

**Figure 4:**
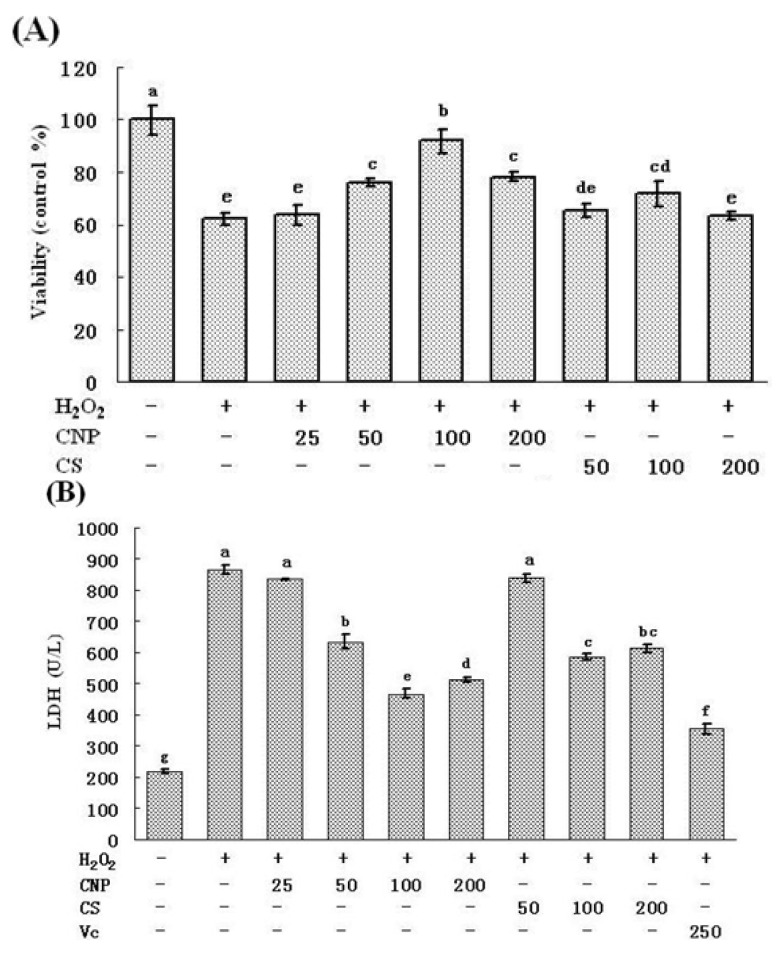
Protective effects of CNP on viability losses in RAW264.7 cells induced by H_2_O_2_ (500 μM). Values are presented as means ± SD. Bars with different letters are statistically different (*P* < 0.05).

Effects of CNP on the viability of H_2_O_2_-induced RAW264.7 cells were evaluated by MTT analysis. The results in Figure 4A show that the survival rate of RAW264.7 cells was decreased markedly (*P* < 0.05) after exposure to 500 μM of H_2_O_2_ for 12 h. However, pre-incubation of RAW264.7 cells with different concentrations of CNP (50, 100, 200 μg/mL) for 24 h significantly increased the vialibity of H_2_O_2_-induced RAW264.7 cells in a dose-dependent manner (*P* < 0.05). In addition, no difference was found in cell viability between cells treated with CNP (25–200 μg/mL) alone and vehicle-treated controls (data not shown). Apparently, CNP were effective for the protection of RAW264.7 cells against H_2_O_2_-induced injury.

To further investigate the protective effects of CNP, LDH assay, another indicator of cell toxicity, was performed. As shown in Figure 4B, LDH release in RAW264.7 cells was minimal in the vehicle-treated control group and a dramatic increase was observed after 12 h exposure to 500 μM of H_2_O_2_ Contrary to this, pre-treatment with CNP for 24 h at concentrations above 50 μg/mL attenuated, markedly, the H_2_O_2_-induced increase in LDH release as well as CS (100, 200 μg/mL), while at the same concentration, CNP significantly decreased LDH release as compared to CS (*P* < 0.05). The results was consistent with that determined by MTT assay.

### 2.4. Effects of CNP on Morphology of RAW264.7 Cells

**Figure 5:**
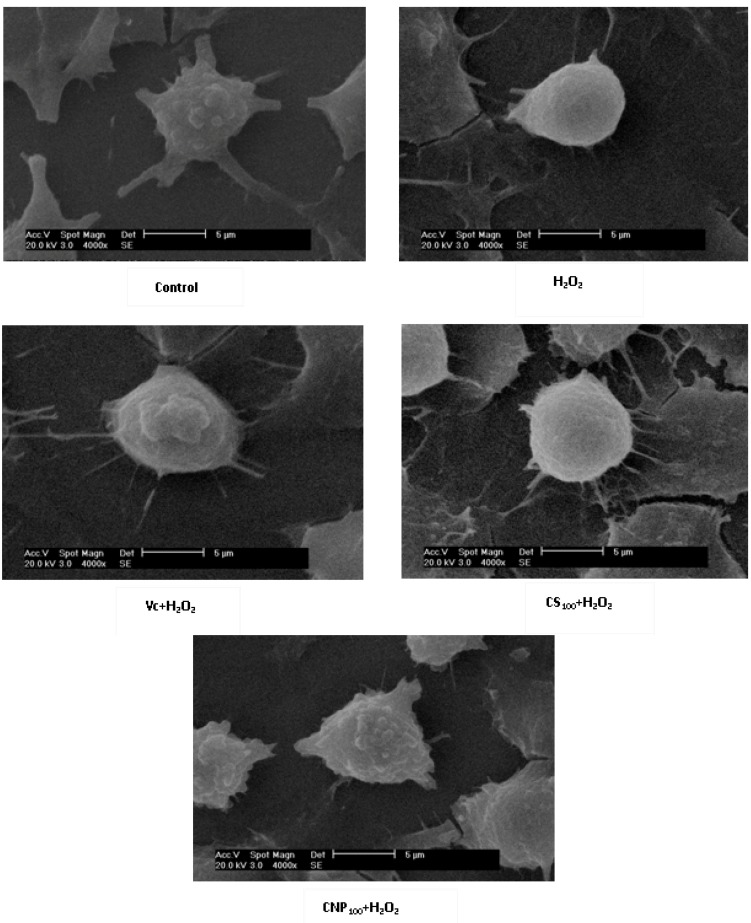
Effects of CNP on morphology of RAW264.7 cells observed with Scanning Electron Microscope (SEM) (4000×).

As shown in Figure 5, RAW264.7 cells treated with CNP (100 μg/mL), Vitamin C, and the vehicle-treated control group exhibited a typical macrophage-like morphology, indicated by the presence of surface ruffles and numerous extending pseudopodia, however, RAW264.7 cells with 500 μM of H_2_O_2_ for 12 h were observed with morphological differences that were indicated by the presence of surface smooth, shrink, and a small number of pseudopodia compared to the vehicle-treated control group.

### 2.5. Measurement of SOD, GSH-Px, GSH, and T-AOC Activities as Well as MnSOD and GSH-Px mRNA Expression Levels

**Figure 6:**
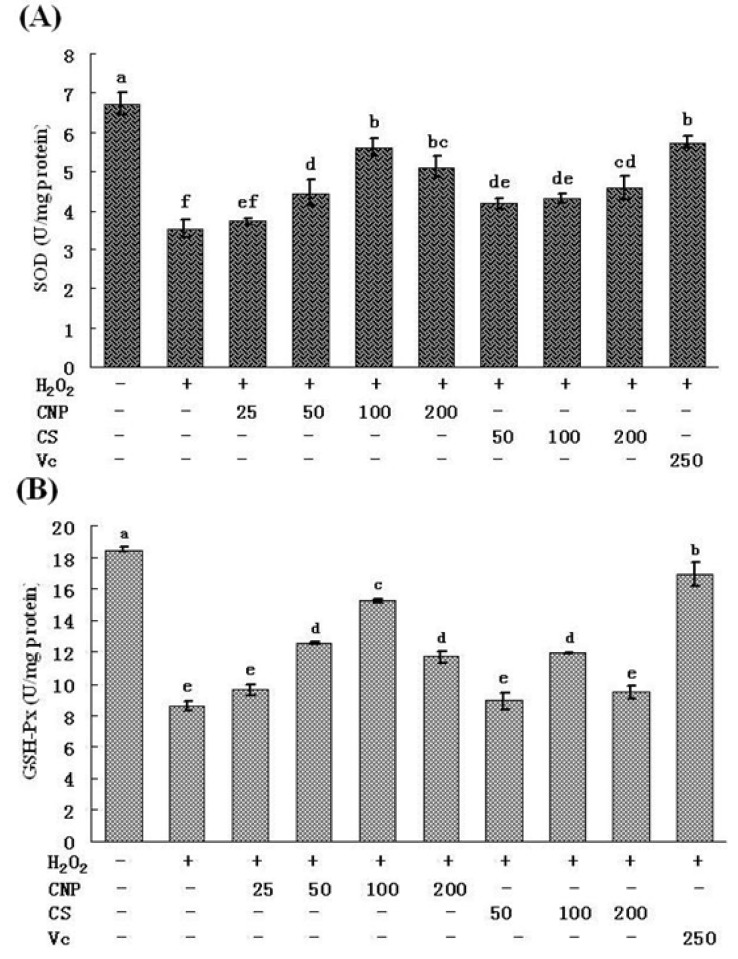
Effects of CNP and antioxidant Vitamin C (Vc) on the production of SOD (**A**), GSH-Px (**B**) in RAW264.7 cells. Values are presented as means ± SD. Bars with different letters are statistically different (*P* < 0.05).

**Figure 7:**
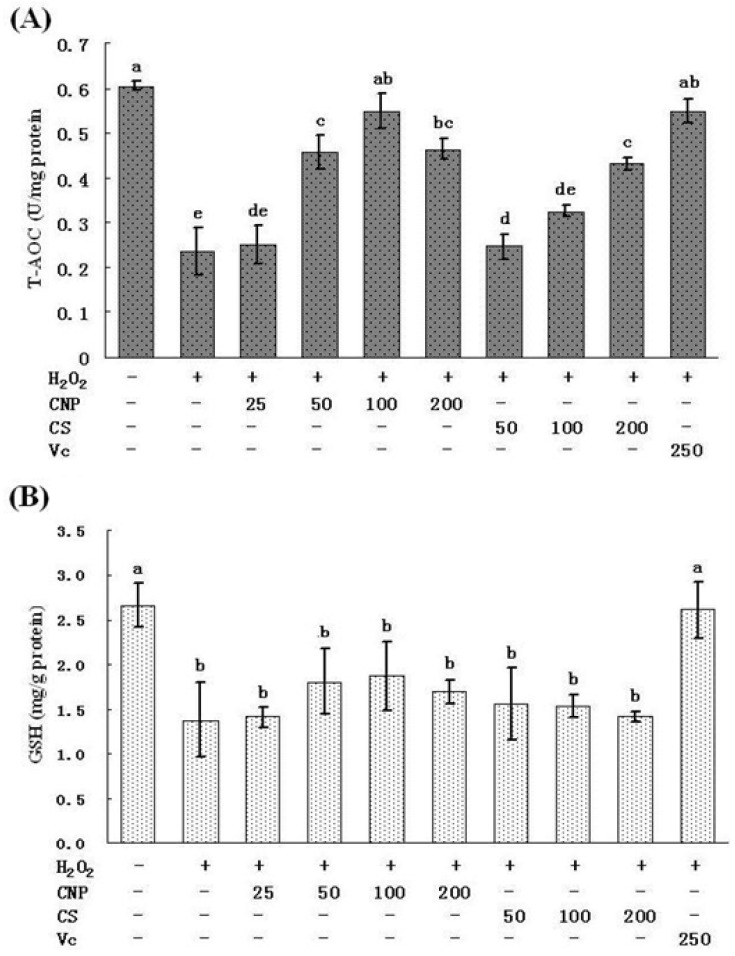
Effects of CNP and antioxidant Vitamin C (Vc) on the production of T-AOC (**A**), GSH (**B**) in RAW264.7 cells. Values are presented as means ± SD. Bars with different letters are statistically different (*P* < 0.05).

Treatment of RAW264.7 cells with 500 μM of H_2_O_2_ for 12 h caused the decrease in the activities of SOD and GSH-Px, respectively (*P* < 0.05). However, pre-incubation with CNP (50–200 μg/mL) significantly attenuated the changes of SOD and GSH-Px activities (Figure 6A,B). At 100 μg/mL of CNP, the H_2_O_2_-induced decrease in SOD and GSH-Px activities were restored respectively, which were the highest among the concentration of CNP and close to that recovered by Vitamin C at 250 μg/mL. CNP and CS did not affect (*P* > 0.05) the concentration of GSH (Figure 7B) in RAW264.7 cells with 500 μM of H_2_O_2_ for 12 h. However, CNP and CS increased significantly the activity of T-AOC (*P* < 0.05) in a dose-dependent manner as compared to the H_2_O_2_ alone control group (Figure 7A). At the same concentration, CNP can more effectively increase SOD, GSH-Px, and T-AOC activities, as compared to CS (*P* < 0.05).

**Figure 8:**
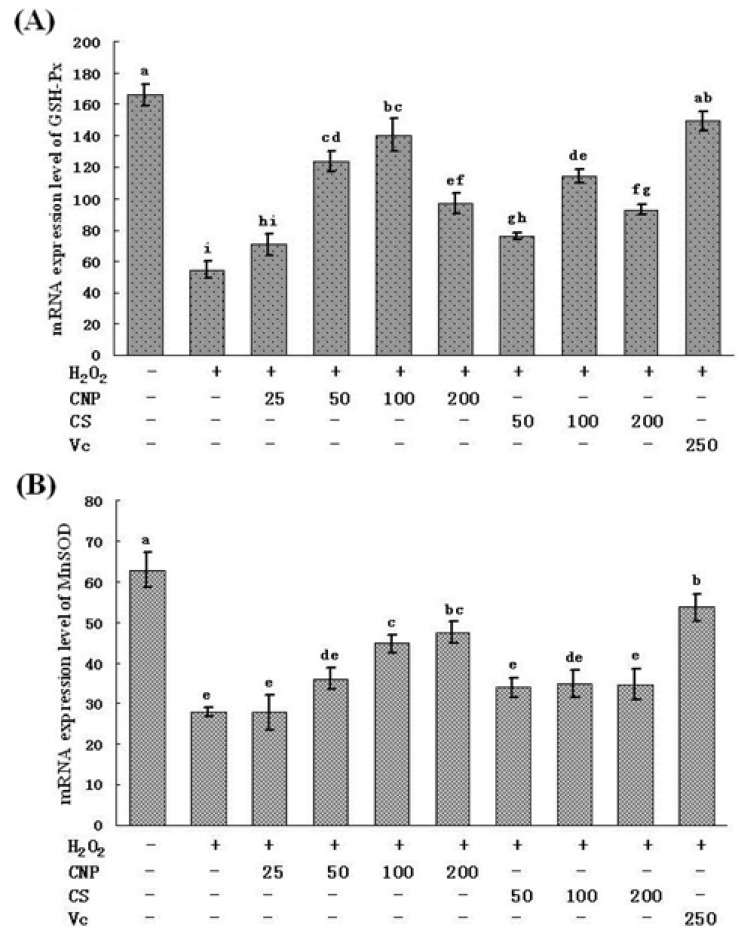
mRNA expression of GSH-Px and MnSOD. Values are presented as means ± SD. Bars with different letters are statistically different (*P* < 0.05).

In parallel, as shown in Figure 8, treatment of RAW264.7 cells with 500 μM of H_2_O_2_ for 12 h caused the decrease in the mRNA expression of MnSOD and GSH-Px. Moreover, after RAW264.7 cells with pre-incubated with CNP (50–200 μg/mL), CS (50–100 μg/mL), or Vitamin C (250 μg/mL) for 24 h, the mRNA expression of SOD and GSH-Px were augmented consistently with intracellular SOD and GSH-Px levels.

### 2.6. Detection of Nitric Oxide (NO) Release in Cell Culture Medium as Well as MDA Content

**Figure 9:**
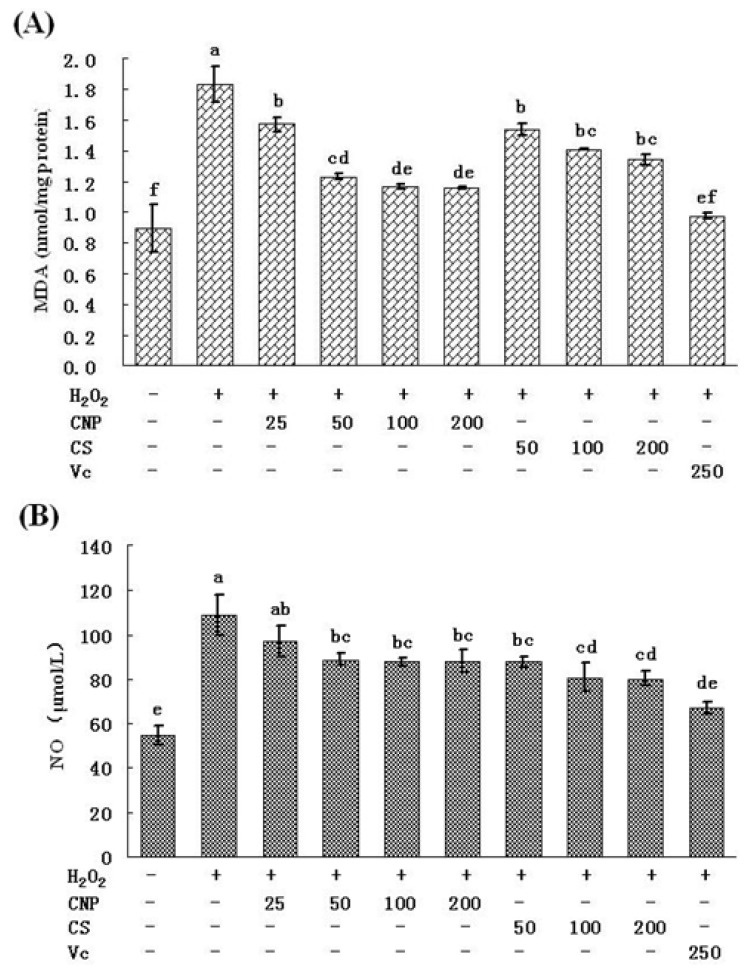
Effects of CNP and antioxidant Vitamin C (Vc) on the production of MDA (**A**), NO (**B**) in RAW264.7 cells. Values are presented as means ± SD. Bars with different letters are statistically different (*P* < 0.05).

RAW264.7 cells treated with 500 μM of H_2_O_2_ for 12 h caused an marked increase in the intracellular MDA level (*P* < 0.05) compared with others, while pre-incubation of cells with CNP (25–200 μg/mL), CS (50–200 μg/mL), or Vitamin C (250 μg/mL) significantly decreased the intracellular MDA level (*P* < 0.05) (Figure 9A), at concentrations (50–200 μg/mL), CNP can more effectively decrease the intracellular MDA level as compared to CS (*P* < 0.05).

In addition, the intracellular NO content in RAW264.7 cells was also assayed according to protocol of commercial kits. As shown in Figure 9B, NO content was increased significantly at the vehicle-treated control group after treatment with 500 μM of H_2_O_2_ for 12 h. In addition pre-incubation with CNP (50–200 μg/mL), CS (50–100 μg/mL), or Vitamin C (250 μg/mL), before H_2_O_2_ exposure, significantly decreased the NO level as compared to H_2_O_2_ treatment alone (*P* < 0.05).

## 3. Discussion

Oxidative stress has been implicated in the pathogenesis of many disease states, such as aging, atherosclerosis, carcinogenesis, ischemia-reperfusing tissue injury, and acute and chronic inflammatory disorders [17,18]. Oxidative stress can be defined as the imbalance between cellular oxidant species production and antioxidant capability. Toxic reactive oxygen species (ROS), including the superoxide and hydrogen peroxide (H_2_O_2_), generated from normal cellular respiration by aerobic metabolism or exogenous oxidants, can cause cellular damage by oxidizing nucleic acids, proteins, and membrane lipids [18]. In biological systems, equilibrium between oxidant formation and endogenous antioxidant defense mechanisms exists to protect cellular biomolecules against oxidation by reactive oxygen species (ROS). ROS are generated excessively in oxidative stress and cause cell or tissue injury, leading to cell death, if this balance is disturbed [19]. Furthermore, ROS have direct or indirect relationships with oxidative process of cellular components and play an important role in various diseases such as cancer, arthritis, inflammation, Alzheimer’s, hypertension, diabetes, and aging [20,21,22].

In this study, we used CNP to pretreat cells before hydrogen peroxide treatment, and cells were washed by PBS, then hydrogen peroxide was used to induce oxidative injury, the uptake of chitosan nanoparticles and hydrogen peroxide were not simultaneous, thus, chitosan nanoparticles did not affect the uptake of hydrogen peroxide. Therefore, we think attenuation of oxidative injury did not depend on inhibition of the uptake of hydrogen peroxide on the cell membrane, maybe due to nanoparticle enhancing efficiency of cellular internalization of chitosan [23]. In addition, there are the previous studies showing that chitosan reduced the oxidative stress *in vivo* and *in vitro* [24,25,26]. Macrophages participate in host defense and are main targets for action of pro-oxidants. H_2_O_2_ is commonly used on macrophage cells for investigation of apoptosis or oxidative stress-mediated cell injury [27,28]. We confirmed that 500 μM H_2_O_2_ and 12 h incubation is sufficient enough to test oxidant-induced cell injury. In this study, we aimed to investigate whether treatment of CNP or CS prior to acute oxidative stress (H_2_O_2_) in macrophage cell can afford to cytoprotection and, if so, if the protection effects of CNP or CS are due to their abilities to enhance cell antioxidant activities. Thus, we examined viability, morphology, the MnSOD, and GSH-Px expression, enzyme and non-enzyme antioxidant activity by CNP on RAW264.7 cells with 500 μM of H_2_O_2_ for 12 h. Current cell culture experiments demonstrated cell viability and morphology were protected significantly by the presence of CNP (50–200 μg/mL). Lactate dehydrogenase (LDH) is a crucial biomarker for assessing cell viability of proliferation [29]. Using LDH as a marker for cell viability and membrane interity, in keeping with viability (MTT assay) and morphology results, this study revealed that pre-treatment of RAW264.7 cells with CNP or CS (50–200 μg/mL) markedly decreased LDH release compared to H_2_O_2_ -induction alone. RAW264.7 cells treated with CNP (100 μg/mL), Vitamin C, and the vehicle-treated control group exhibited a typical macrophage-like morphology, indicated by the presence of surface ruffles and numerous extending pseudopodia, however, RAW264.7 cells with 500 μM of H_2_O_2_ for 12 h were observed morphological differences that indicated by the presence of surface smooth, shrink, and a small number of pseudopodia compared to the vehicle-treated control group. Based on viability and morphology, our findings suggest RAW264.7 cells treated with CNP can effectively attenuate H_2_O_2_-induced cell injury.

Lipid peroxidation is one of the primary events in free radical-mediated cell injury [30]. GSH-Px and SOD are the major antioxidant enzymes, which can effectively eliminate free radicals (FR), and have antioxidative stress function, and they not only prevent the damage of oxygen free radicals, but also have an intrinsic protective effect, therefore maintaining a healthy balance between oxidants and antioxidants [31]. MDA is the direct products of lipid peroxidation after unsaturated fatty acids attacked by radicals on cell membrane and is widely used as a biomarker of oxidative stress [32]. MDA content serves as an indicator of the extent of lipid peroxidation and an indirect reflection of the extent of cell damage. MDA may destruct cell membrane structure, cause DNA fragmentation, rearrangement, cross-linking, and accelerate apoptosis, and is one of the most important elements of phlegmnosis or tumorigenesis [33]. Therefore, determination of MDA, SOD, and GSH-Px can reflect the level of metabolism of oxygen free radicals. On the other hand, cells are often equipped with several antioxidants for the prevention of free-radical damage. SOD and GSH-Px, along with other enzymatic and non-enzymatic antioxidants, play pivotal roles in preventing cellular damage caused by ROS [34]. The MnSOD isoform (SOD2) is primarily located in mitochondria and is considered the primary isoform in relation to oxidative stress [35]. Therefore, the intracellular ROS can be effectively eliminated by the combined action of SOD, GSH-Px, and other endogenous antioxidants, which provide a repairing mechanism for oxidized membrane components. In the present study, significant decreases in SOD and GSH-Px were observed in RAW264.7 cells after exposure to H_2_O_2_, indicating the impairment in antioxidant defenses. Moreover, the mRNA expression of SOD and GSH-Px were found also marked decreases. In addition, an obvious elevation of MDA production was associated with an increase of LDH release. Nonetheless, when RAW264.7 cells were pre-incubated with CNP, these H_2_O_2_-induced cellular events were blocked to a great extent. Our studies also show a similar efficiency in protecting RAW264.7 cells against H_2_O_2_-induced oxidative injury between CNP (50–200 μg/mL) and Vitamin C (250 μg/mL). These results jointly suggest that enhancement of endogenous antioxidant preservation and attenuation of lipid peroxidation may represent a major mechanism of cellular protection by CNP.

NO is one of the most important mediators in the regulation of cell functions [36]. NO stimulates H_2_O_2_ production from the mitochondrial respiratory chain, and NO directly inhibits catalase [37], while peroxynitrite oxidizes glutathione and inactivates glutathione reductase [38]. Thus, NO can raise H_2_O_2_ levels in cells. H_2_O_2_ and NO can react on superoxide dismutase to produce peroxynitrite [39]. Thus, high NO levels in cell can, in the presence of oxygen, give rise to a potent mixture of ROS and reactive nitrogen species (RNS), and the most significant results of this are oxidation of protein cysteine residues, lipid oxidation, and DNA mutation [40]. In this study, we found CNP could significantly decrease NO level as compared to the H_2_O_2_ alone control group, the result suggest that CNP can attenuate RAW264.7 cells injury by H_2_O_2_-induction, which may be through decrease in NO content.

## 4. Experimental Section

### 4.1. Chemicals and Reagents

Dulbecco’s modified Eagle’s medium (DMEM), penicillin/streptomycin, and the other materials required for culture of cells were purchased from Gibco BRL, Life Technologies (Grand Island, NY, USA). H_2_O_2_, dimethylsulfox-ide (DMSO), 3-(4,5-dimethylthiazol-2-yl) 2,5-diphenyltetrazolium bromide (MTT), Vitamin C, and bovine serum albumin (BSA) were obtained from Sigma (St. Louis, MO, USA). The reagent kits for measurement of levels of LDH, NO, MDA, SOD, GSH-Px, GSH, and T-AOC were purchased from the Nanjing Institute of Jiancheng Bioengineering (Nanjing, China). Trizol was from Invitrogen (Carlsbad, CA, USA), revert Aid™ M-MuLV reverse transcriptase was from Fermentas (Amherst, NY, USA), diethylpyrocarbonate (DEPC) and ribonuclease inhibitor were from Biobasic, Canada, oligo (dT)_18_ were from Sangon, China. All other chemicals were of analytical grade or of the highest grade available commercially.

### 4.2. Cell Culture and Treatment

Mouse macrophages RAW 264.7 cell line was obtained from the Shanghai Institute of Cell Biology (Shanghai, China) and maintained in DMEM, supplemented with heat-inactivated 10% fetal bovine serum, 100 U/mL penicillin, 100 U/mL streptomycin in a humidified atmosphere of 5% CO_2_ at 37 °C. When the cells reached sub-confluence, they were pretreated for 24 h with culture medium containing different concentrations of CNP (25, 50, 100, and 200 μg/mL), CS (50, 100, and 200 μg/mL), or Vitamin C (250 μg/mL) that were tested in the experiments. Following this, the culture supernatant was collected and cells were washed three times with phosphate-buffered saline (PBS, pH 7.2). Subsequently, the cells were exposed to H_2_O_2_ (500 μM) diluted in culture medium for 12 h in a humidified atmosphere of 5% CO_2_ at 37 °C until further assay.

### 4.3. Preparation and Characterization of CNP

Chitosan (CS) was obtained from the Chitosan Company of Pan’an (Zhejiang, China). The degree of deacetylation was about 95%, as determined by elemental analysis, and the average molecular weight of the chitosan was 220 kDa, as determined by viscometric methods. Chitosan nanoparticles were prepared and characterized as described previously [14,16]. Briefly, Chitosan was dissolved at 0.5% (w/v) with 1% (v/v) acetic acid (HOAc) and then raised to pH 4.6–4.8 with 10 N NaOH. CNP were formed by coacervation between positively charged chitosan (0.5%, w/v) and negatively charged sodium tripolyphosphate (0.25%, w/v). Nanoparticles with different mean size were obtained by adjusting the volume ratio of chitosan to tripolyphosphate solution. Nanoparticles were purified by centrifugation at 9000× *g* for 30 min. Supernatants were discarded and chitosan nanoparticles were extensively rinsed with distilled water to remove any NaOH residue, and freeze dried before further use or analysis. The freeze-dried chitosan nanoparticles were suspended in water for characterization or use for other experiments. Particle size distribution and the zeta potential of chitosan nanoparticles were determined using Zetasizer Nano-ZS90 (Malvern, Worcestershire, UK). The analysis was performed at a scattering angle of 90° at a temperature of 25 °C using samples diluted to different intensity concentration with de-ionized distilled water. Atomic force microscopy (AFM, SPM-9500J3) was used for visualization of the chitosan nanoparticles deposited on silicon substrates operating in the contact mode. AFM imaging was performed using Si3N4 probes with a spring constant of 0.06 N/m.

### 4.4. Cell Viability Measurement

RAW 264.7 cells were seeded at density of 1 × 10^6^ cells/mL in 6-well plates and the cell viability was measured using the MTT assay [41]. Briefly, at the indicated time after the treatment as before, the culture supernatant was removed. The cells were washed with PBS and incubated with MTT (5 mg/mL) in culture medium at 37 °C for another 4 h. After MTT removal, the colored formazan was dissolved in 150 μL of DMSO. The absorption values were measured at 490 nm using a SpectraMax M5 Microplate Reader (Molecular Devices, MDS Analytical Technologies, Sunnyvale, CA, USA). The viability of RAW 264.7 cells in each well was presented as percentage of control cells.

### 4.5. Morphology of RAW264.7 Cells

RAW264.7 cells (1 × 10^6^ per coverslip) were seeded onto sterile coverslips and treated as the same as “*4.2. Cell Culture and Treatment*” in a humidified CO_2_ incubator at 37 °C. Following incubation, cells were prepared for scanning electron microscopy. Briefly, coverslip cultures were rinsed with PBS, fixed in 2.5% glutaraldehyde at 4 °C overnight, and post-fixed in 1% osmium tetroxide for 1 h. Coverslip cultures, then, were washed in 0.1 M sodium cacodylate, dehydrated in a graded series of ethanol 50%, 70%, 80%, and 95%, 3×, 10 min each; followed by 100%, 3×, 20 min each and immersed in hexamethyldisilazane (HMDS, 3×, 15 min each). Coverslip cultures were air-dried, mounted, and coated with gold. Cells were examined using a XL30-ESEM Scanning Electron Microscope operating at an accelerating voltage of 20 kV.

### 4.6. Preparation of Cell Lysates

The cells were seeded at a density of 1 × 10^6^ cells/mL in 6-well plates. When the cells reached sub-confluence, they were pretreated for 24 h with culture medium containing different concentrations of CNP (25, 50, 100, and 200 μg/mL), CS (50, 100, and 200 μg/mL), or Vitamin C (250 μg/mL). Upon completion of the incubation studies, the culture supernatant was collected for analysis of LDH and NO release. The cells were scraped from the plates into ice-cold 1% Triton X-100 lysis buffer and protein concentration was determined by the bicinchoninic acid (BCA) method, using BSA as a reference standard. Aliquots were stored at −80 °C until detection for MDA, SOD, GSH-Px, GSH, and T-AOC.

### 4.7. Measurement of LDH and Nitric Oxide (NO) Release

LDH, an indicator of cell injury, was detected after the exposure to H_2_O_2_ with an assay kit according to the manufacturer’s protocol. The activity of enzyme was expressed as units per liter and the absorbance was read at 450 nm. The concentration of nitriles (NO_2_^−^) and nitrates (NO_3_^−^), stable end products of nitric oxide (NO), were determined by the reagent kits from the Nanjing Institute of Jiancheng Bioengineering (Nanjing, China). NO production was determined by measuring the optical density at 550 nm and expressed as units per liter.

### 4.8. Assay for Intracellular Contents of SOD, GSH-Px, and MDA

The activities of SOD and GSH-Px, as well as the concentration of MDA, were all determined by using commercially available kits. All procedures completely complied with the manufacturer’s instructions. The activities of enzymes were expressed as units per milligram protein. The assay of SOD activity was based on its ability to inhibit the oxidation of hydroxylamine by O^2−^ produced from the xanthine-xanthineoxiase system. One unit of SOD activity was defined as the amount that reduced the absorbance at 450 nm by 50%. The assay of GSH-Px activity was assayed by quantifying the rate of oxidation of the reduced glutathione to the oxidized glutathione by H_2_O_2_ catalyzed by GSH-Px. One unit of GSH-Px was defined at 412 nm as the amount that reduced the level of GSH by 1 μM in 1 min/mg protein. MDA was measured at a wavelength of 532 nm by reacting with thiobarbituric acid (TBA) to form a stable chromophoric production. Values of MDA level were expressed as nanomoles per milligram protein.

### 4.9. Measurement of the mRNA Expression Levels of MnSOD and GSH-Px by Real-Time PCR

Cells were lysed in 0.8 mL of Trizol reagent (Invitrogen™, Carlsbad, CA, USA) and the total RNA was isolated according to the manufacture’s protocol. The concentration of total RNA was quantified by determining the optical density at 260 nm. The total RNA was used and reverse transcription was performed by mixing 2 µg of RNA with 0.5 µg oligo (dT)_18_ primer in a DEPC-treated tube. Nuclease-free water was added giving a final volume of 12.5 µL. This mixture was incubated at 70 °C for 5 min and chilled on ice for 2 min. Following this, a solution containing 4 µL of M-MuLV 5× reaction buffer, 2 µL of 10 mM dNTP, 20 U of ribonuclease inhibitor, and DEPC-treated water was added, giving a final volume of 19 μL, and the tubes were incubated for 5 min at 37 °C. The tubes then received 200 U of M-MuLV reverse transcriptase and were incubated for 60 min at 42 °C. Finally, the reaction was stopped by heating at 70 °C for 10 min. The samples were stored at −20 °C until further use.

As shown in Table 1, the primers were used to amplify cDNA fragments (238-bp GSH-Px fragment, 79-bp MnSOD fragment, and 94-bp 18S fragment). Amplification was carried out in total volume of 20 µL containing 1 µL (5 µM) of each target and 18S specific primers, 2 µL of cDNA template, 2 µL of 10× PCR buffer, 2 μL of MgCl_2_ (25 mM), 2 μL of dNTPs (2.5 mM), 2 μL of transcribed cDNA, and 0.4 μL of Taq DNA polymerase. Reaction conditions were the standard conditions for the iQTM5 PCR (Bio-Rad, Hercules, CA, USA) (10 s denaturation at 95 °C, 25 s annealing at 60 °C (GSH-Px, MnSOD), 62 °C (18S)) with 45 PCR cycles. Ct values were obtained automatically using software (Bio-Rad, USA). The comparative Ct method (2^−ΔΔCt^ method) [42] was used to analyze the expression levels of MnSOD and GSH-Px.

**Table 1:** Real-Time PCR Primers and Conditions.

Gene	Genbank Accession	Primer sequence	Product size (bp)	Annealing (°C)
GSH-Px	NM_008160	5′ ACAGTCCACCGTGTATGCCTTC 3′	238	60
		5′ CTCTTCATTCTTGCCATTCTCCTG 3′		
MnSOD	X04972	5′ CTGTGGGAGTCCAAGGTTCA 3′	79	60
		5′ GAGCAGGCAGCAATCTGTAAG 3′		
18S	NR_003278	5′ CGGACACGGACAGGATTGACA 3′	94	62
		5′ CCAGACAAATCGCTCCACCAACTA 3′		

### 4.10. Effect of CNP on the Antioxidant Capacity of H_2_O_2_-Induced RAW264.7 Cells

Levels of GSH were determined colorimetrically at 405 nm with the spectrophotometer following reaction with 5,5-dithiobes (2-ni-trobenzoic acid) (DTNB), and was expressed as mg gpro^−1^. T-AOC was measured according to protocol of commercial kits. In the reaction mixture ferric ion was reduced by antioxidant reducing agents and to form the complex ferrous-tripyridyltriazine, which can be estimated by colorimetric assay to reflect T-AOC, which was expressed as U mgpro^−1^.

### 4.11. Statistical Analysis

Data were expressed as mean ± standard deviations (S.D.) and examined for their statistical significance of difference with ANOVA and a Tukey *post hoc* test by using SPSS 16.0. *P*-values of less than 0.05 were considered statistically significant.

## 5. Conclusions

The present studies clearly show that chitosan nanoparticles (CNP) can attenuate H_2_O_2_-induced stress injury in RAW264.7 cells, and the unique character of nanoparticles could make CNP exhibit more superior antioxidative activities than chitosan (CS). We explored the protective mechanism of action of CNP against H_2_O_2_-induced RAW264.7 cell injury, which are partly contributed to restoring the activities endogenous antioxidants, along with enhancement of their gene expression. These data propose a possibility that CNP can be used as a potent natural antioxidant in the treatment of oxidative-related diseases. Since oxidative stress-induced RAW264.7 cell injury plays a key role in inflammation disease, our findings suggest a novel application for CNP in the treatment of chronic inflammation.
